# Gene dosage effects in yeast support broader roles for the *LOG1*, *HAM1* and *DUT1* genes in detoxification of nucleotide analogues

**DOI:** 10.1371/journal.pone.0196840

**Published:** 2018-05-08

**Authors:** Mattias Carlsson, Guo-Zhen Hu, Hans Ronne

**Affiliations:** Department of Forest Mycology and Plant Pathology, Swedish University of Agricultural Sciences, Uppsala, Sweden; Texas A&M University, UNITED STATES

## Abstract

Purine and pyrimidine analogues have important uses in chemotherapies against cancer, and a better understanding of the mechanisms that cause resistance to these drugs is therefore of importance in cancer treatment. In the yeast *Saccharomyces cerevisiae*, overexpression of the *HAM1* gene encoding inosine triphosphate pyrophosphatase confers resistance to both the purine analogue 6-N-hydroxylaminopurine (HAP) and the pyrimidine analogue 5-fluorouracil (5-FU) (Carlsson et al., 2013, PLoS One *8*, e52094). To find out more about the mechanisms of resistance to nucleotide analogues, and possible interdependencies between purine and pyrimidine analogue resistance mechanisms, we screened a plasmid library in yeast for genes that confer HAP resistance when overexpressed. We cloned four such genes: *ADE4*, *DUT1*, *APT2*, and *ATR1*. We further looked for genetic interactions between these genes and genes previously found to confer resistance to 5-FU. We found that *HMS1*, *LOG1 (YJL055W)*, *HAM1*, and *ATR1* confer resistance to both 5-FU and HAP, whereas *ADE4*, *DUT1* and *APT2* are specific for HAP resistance, and *CPA1* and *CPA2* specific for 5-FU resistance. Possible mechanisms for 5-FU and HAP detoxification are discussed based on the observed genetic interactions. Based on the effect of *LOG1* against both 5-FU and HAP toxicity, we propose that the original function of the LOG (LONELY GUY) family of proteins likely was to degrade non-canonical nucleotides, and that their role in cytokinin production is a later development in some organisms.

## Introduction

Antimetabolite drugs such as purine and pyrimidine analogues play an important role in chemotherapy against cancer. However, tumours may acquire resistance to such drugs by clonal selection of resistant cancer cells. A better understanding of the mechanisms of action of anticancer drugs and in particular the ways by which drug resistance can arise is therefore of importance both for cancer treatment and for the development of new more efficient anticancer drugs. Since nucleotide metabolism is evolutionarily conserved, these mechanisms can be studied in model organisms such as *Saccharomyces cerevisiae* (baker’s yeast) where advanced methods for molecular genetics are available.

The first antimetabolite drug that was developed to specifically target cancer cells was the pyrimidine analogue 5-fluorouracil, 5-FU [1]. 5-FU targets several cellular mechanisms through its activated metabolites FdUMP, FUTP and FdUTP [2–3]. FdUMP inhibits thymidylate synthase (TYMS), and this is a major mechanism behind both the toxicity and the anticancer effect of 5-FU [4–8]. However, effects on RNA metabolism also play an important role in 5-FU toxicity [9–12]. Such effects result from inhibition of RNA modifications such as methylation and pseudouridylation [11,13], which in turn cause disturbed exosome processing of polyadenylated rRNA [14], interference with spliceosome function [15], and destabilization of tRNAs [12].

We previously carried out a screen in yeast for genes that confer resistance to 5-FU when overexpressed from a high copy number plasmid [16]. Among the cloned resistance genes we found *HAM1*, which encodes inosine triphosphate pyrophosphatase, an enzyme that dephosphorylates non-canonical purine nucleoside triphosphates ITP, dITP and XTP and thus prevents their incorporation into DNA and RNA [17–18]. This finding suggested that the Ham1 protein may have a broader specificity than originally thought, targeting non-canonical pyrimidines in addition to purines [16]. It also raised questions about interactions between the purine and pyrimidine metabolic pathways and the role of such interactions in acquired resistance to nucleotide analogues.

To look further into these questions, we decided to carry out a screen for yeast genes that confer resistance to purine analogues when overexpressed. For this screen we chose to use 6-N-hydroxylaminopurine (HAP), the drug that was originally used to identify *HAM1* as a gene that causes hypersensitivity to purine analogues when disrupted [17]. HAP is a cytotoxic and hemolytic purine analogue that is similar to adenine but with a hydroxylamine group instead of an amino group attached to the number 6 carbon. In contrast to other purine analogues such as azathioprine and mercaptopurine [19–20] it is not used in medicine since it is hemolytic at concentrations below what could have therapeutic potential [21]. However, it has been used in research to study purine analogue toxicity in both human tumor cell lines and in the yeast *Saccharomyces cerevisiae* [17,22–25].

The screen was carried out both in a wild type strain and in a *ham1* knockout mutant that is hypersensitive to HAP. We found four new yeast genes that confer resistance to HAP when overexpressed: *ADE4*, *DUT1*, *APT2*, and *ATR1*. We proceeded to test these genes for their effects on 5-FU resistance. We also looked for genetic interactions between the genes isolated in the 5-FU and HAP resistance screens as well as interactions with other genes involved in purine metabolism. Based on our findings, we discuss possible roles of the cloned genes in the metabolism and detoxification of HAP, 5-FU and other nucleotide analogues. In particular, we propose that the original function of the widely distributed LOG (LONELY GUY) family of proteins was to facilitate the removal of non-canonical nucleotides from the nucleotide pool, working downstream of Ham1p and other nucleotide phosphatases, and that their role in cytokinin production in plants and some microorganisms [26–28] is a later development that occurred in these organisms.

## Materials and methods

### Yeast strains and plasmids

Yeast deletion strains in the BY4742 haploid and the isogenic BY4743 diploid background were obtained from Euroscarf (http://www.uni-frankfurt.de/fb15/mikro/euroscarf). The open reading frame in each deletion strain has been replaced by the *KanMX* selection cassette [29]. The plasmids pCPA1, pCPA2, pHMS1, pYJL055w, pHAM1 have been described previously [16]. Plasmid pYJL055w is referred to as pLOG1 in the present paper in order to make the genetic nomenclature consistent.

### PCR cloning

Plasmid pAPT1 was constructed by PCR amplification of a fragment spanning from 430 bp upstream to 150 bp downstream of the *APT1* ORF, with primers adding a *Sac*I site at the 3’ end: 5'-*GAG CTC* GCA CTC CAG AAA CAA CAG CA-3', and a *BamH*I site at the 5’ end: 5'-*GGA TCC* TGT GGC ACA AAG CAG AAA AG-3'. The PCR fragment was TA-cloned into pCR2.1 (Invitrogen, US) and subsequently subcloned between the *Sac*I and *BamH*I sites in the polylinker of the shuttle vector pHR81 [30]. Plasmid pATR2 was constructed by PCR amplification of a fragment spanning from 356 bp upstream to 210 bp downstream of the *ATR2* ORF, with primers adding *Sac*I sites at both ends: 5'-*GAG CTC* ACA GGG GTG CGC ATA AAT AG-3' and 5'-*GAG CTC* CTT GCG CAA ATG AAG AAC AA-3'. The PCR fragment was TA-cloned into pCR2.1, and subsequently subcloned into the *Sac*I site in the polylinker of pHR81.

### Growth media and chemicals

Rich media (YPD) and synthetic complete media (SC) or dropout media based on SC were prepared as previously described [31]. The synthetic media contained either 2% glucose or 2% galactose as carbon source. 6-N-Hydroxylaminopurine (HAP) and 5- fluorocytosine (5-FC) were obtained from Apollo Scientific (Manchester, UK). 5-Fluorouracil (5-FU) and 6-azauracil (6-AzaU) were obtained from Sigma-Aldrich (Stockholm, Sweden). 8-azaguanine (8-AzaG) was obtained from Accel Pharmatech (East Brunswick, US). Boric acid was obtained from Fluka Chemie (Buchs, Switzerland).

### Shuttle plasmid library screen

**Fig 1:**
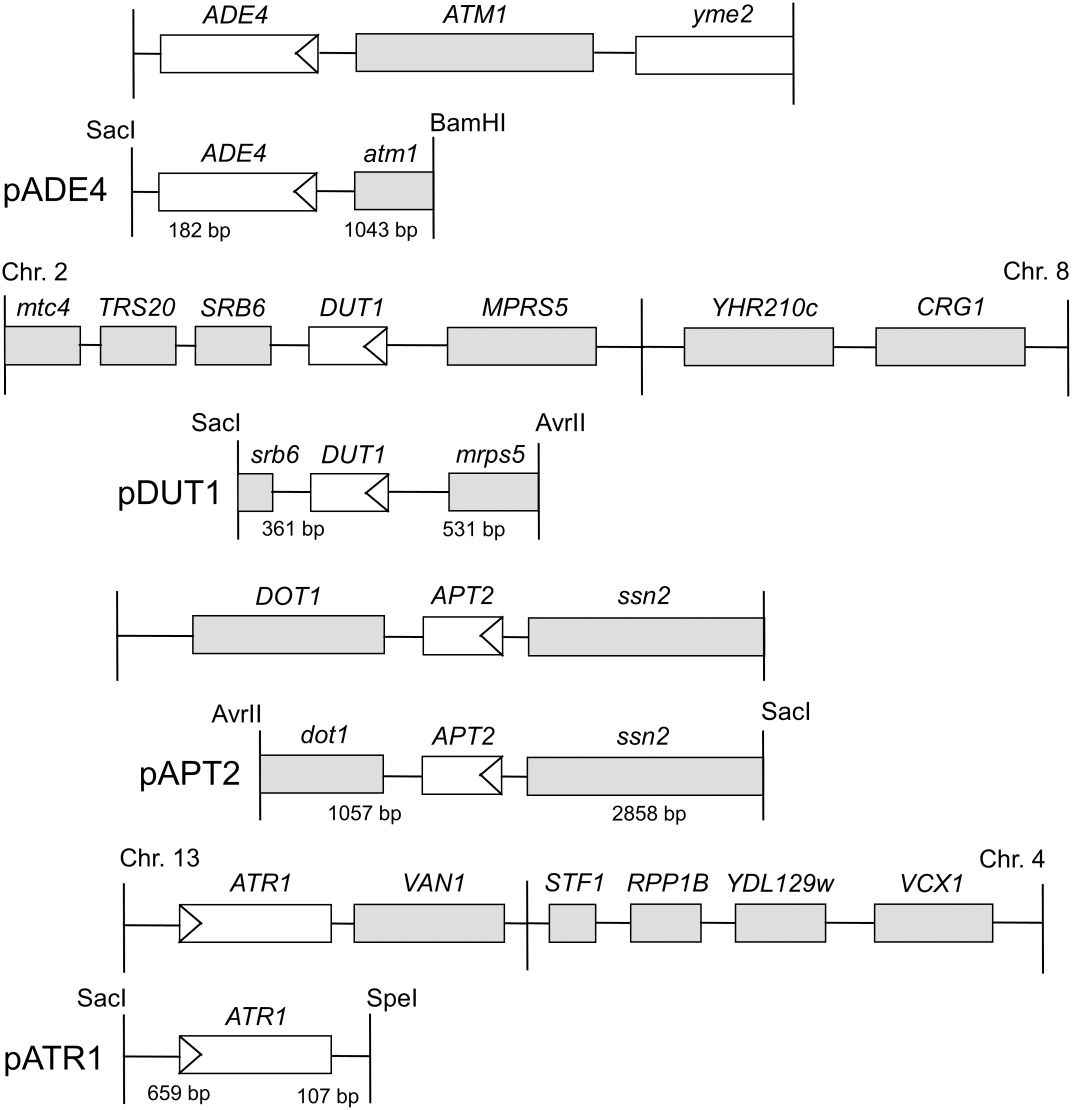
Restriction maps of plasmids isolated in the HAP resistance screen. The shortest subclone that could still confer HAP resistance when overexpressed is shown below each plasmid. Open reading frames are shown as grey boxes, with the mapped resistance gene as a white box. The numbers of included base pairs upstream and downstream of the open reading frame in each minimal subclone are also shown.

The BY4742 wild type and the *ham1* knockout strain were transformed with a yeast genomic library made in the 2 μm *URA3 LEU2-d* vector pHR81 [30]. The copy number of 2 μm plasmids is 5–50 molecules per cell, and will adjust if the insert is selected for or against. It is therefore possible to recover also weak dosage suppressors in a library screen which may require a higher copy number to be effective. To select for HAP resistance, the wild type transformants were plated on SC galactose media without uracil and adenine containing 50 μg/ml HAP, and the *ham1* transformants were plated on SC glucose media without uracil and adenine containing 50 μg/ml HAP. Early emerging colonies and colonies with greater size were picked to grids on drug free media. In order to verify that the picked transformants were HAP resistant, they were sequentially replicated twice to the same HAP containing media that they were initially selected on. Plasmids were rescued from confirmed HAP resistant colonies, retransformed into the wild type BY4742, and retested for HAP resistance. The genes responsible for HAP resistance were mapped by deletions and PCR subcloning (Fig 1), followed by testing of the resulting plasmids after retransformation into yeast. We estimate that in total we screened approximately 120 000 transformants.

### Yeast growth and spot assays

To assay drug sensitivity, transformants were grown overnight at 30 °C in SC galactose or glucose media without uracil. These overnight precultures were diluted into fresh media to a final OD_600_ of 0.1 and grown to late exponential phase. Cultures were diluted to OD_600_ 0.1 in water before spotting. For the overexpression and disruption assays 10-fold serial dilutions were made. A 2.5 μl aliquot of each dilution was spotted onto control plates and drug plates. The drug concentrations used were higher than in the initial screens since we wanted to highlight differences between different suppressor plasmids. Growth was monitored daily starting after two days at 30 °C.

## Results

### Cloning of genes that confer resistance to HAP when overexpressed

In order to identify yeast genes that confer resistance to purine nucleotide analogues when overexpressed we screened a yeast genomic DNA library in the high copy number shuttle vector pHR81 [30] for plasmids that confer resistance to HAP when overexpressed. The screen was carried out both in a wild type strain and in a *ham1* knockout mutant that is hypersensitive to HAP in order to facilitate the recovery of weaker resistance genes. Transformants in the wild type strain were screened on HAP-containing media with galactose as a carbon source since we found that galactose increases the sensitivity to HAP, whereas transformants in the hypersensitive *ham1* mutant were screened on HAP-containing media with glucose as a carbon source. The reason for this difference remains to be determined, but it is not unusual to see phenotypic differences between cells grown on glucose and other carbon sources, since glucose repression is a global regulatory response that alters the expression of a large part of the yeast genome [32].

**Fig 2:**
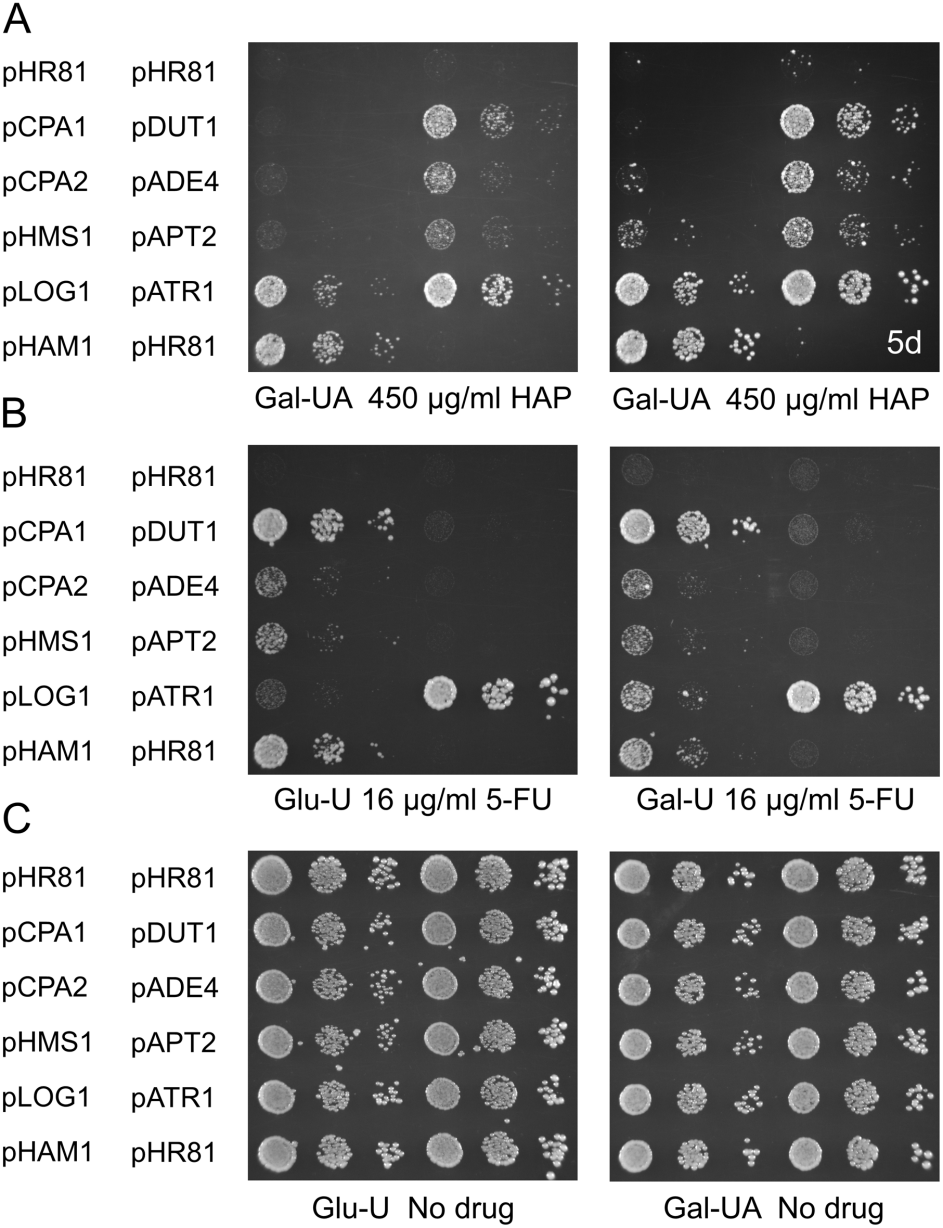
Resistance to HAP and 5-FU due to overexpression of different genes. The genes isolated in our HAP resistance screen were tested for resistance to HAP (A) and 5-FU (B). No drug controls are shown in panel C. Also included were the genes isolated in our previous screen for 5-FU resistance [16]. Cells transformed with the empty vector pHR81 [30] were included as a negative control. Transformants were grown in liquid medium to late exponential phase, serially diluted, and then spotted onto uracil- and adenine-less SC galactose (Gal-UA) plates, uracil-less glucose (Glu-U) or uracil-less galactose (Gal-U) plates with or without HAP or 5-FU at the indicated concentrations. Plates were incubated for 3 days except for the second plate containing HAP, which was incubated for 5 days (5d) in order to show the weak effect of *HMS1* overexpression.

In total, we screened approximately 120 000 transformants, which corresponds to a 30-fold coverage of the yeast genome, assuming an average insert size of 4 kbp. After rescue of the plasmids back into *E*. *coli*, retransformation into yeast for confirmation of the HAP resistance phenotype, and mapping of the HAP resistance within the plasmid inserts, we identified four genes, *ADE4*, *DUT1*, *APT2*, and *ATR1*, that confer resistance to HAP when overexpressed (Fig 1). *ADE4*, *DUT1* and *APT2* were cloned from the *ham1* strain, whereas *ATR1* was cloned from the wild type. In addition, the *HAM1* gene was isolated four times from the *ham1* strain and once from the wild type. The resistance conferred by each cloned gene to HAP is shown in Fig 2A. As discussed below, some of the genes also confer resistance to 5-FU (Fig 2B).

*ADE4* encodes 5-phosphoribosyl-1-pyrophosphate amidotransferase, which catalyzes the first step in the *de novo* synthesis of purine nucleotides. Overexpression of *ADE4* causes increased synthesis of purine nucleotides and has been shown to mediate resistance to the DNA-crosslinker cisplatin, which is used as an anticancer drug [33]. We found that *ADE4* overexpression confers resistance to HAP (Fig 2A) but not to 5-FU (Fig 2B), which is consistent with a model where increased *de novo* synthesis of purines suppresses the toxicity of HAP by diluting the drug with freshly synthesized purines.

*DUT1* is an essential gene encoding deoxyuridine triphosphate diphosphatase (dUTPase), an enzyme required both for *de novo* synthesis of thymidylate, and for genome stability by preventing incorporation of uridylate into DNA [34]. *DUT1* is also known to be important for the resistance to antifolates such as aminopterin and the anticancer drug methotrexate [35]. Since Dut1p is thought to act primarily on dUTP, a pyrimidine nucleotide, one might expect it to confer resistance to 5-FU when overexpressed. However, we found that whereas *DUT1* clearly confers resistance to HAP (Fig 2A), its effect, if any, on 5-FU resistance is barely detectable (Fig 2B, galactose).

**Fig 3:**
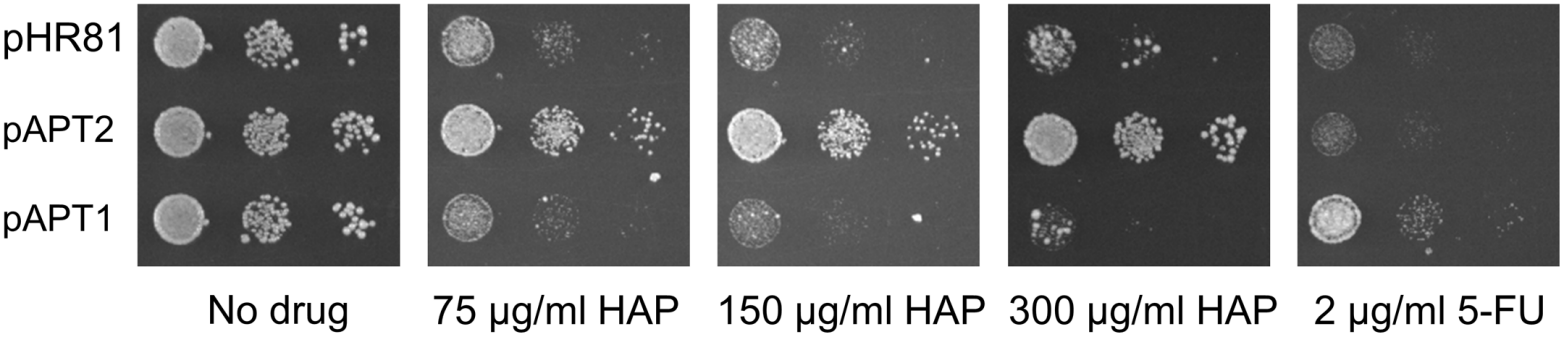
Resistance to HAP and 5-FU conferred by overexpression of *APT2* and *APT1*. The cloned *APT2* plasmid and a PCR-amplified *APT1* gene cloned into the vector pHR81 were tested for resistance to HAP and 5-FU. Cells transformed with pHR81 [30] were included as a negative control. Transformants were grown in liquid medium to late exponential phase, serially diluted, and then spotted onto uracil- and adenine-less SC galactose (Gal-UA) plates with or without HAP or 5-FU at the indicated concentrations.

*APT2* is a duplicated copy of the *APT1* gene encoding adenine phosphoribosyltransferase (APRT). *APT1* and *APT2* were identified as one of 549 gene pairs that remain as evidence of an ancient whole genome duplication in the *Saccharomyces* lineage [36]. The retention of both genes suggests that they may have acquired unique and different functions. However, Apt2p lacks APRT activity when expressed in *E*.*coli* and a disruption of the *APT2* gene has no apparent phenotype in yeast [37]. It is thus not clear what enzymatic activity, if any, that Atp2p possesses. Nevertheless, we found that the *APT2* gene confers resistance to HAP (Fig 2A) but not to 5-FU (Fig 2B) when overexpressed. We also tested a PCR clone of *APT1* (Fig 3). As expected, it did not confer resistance to HAP, but instead had a slightly poorer growth on HAP. However, it did instead confer some resistance to 5-FU at low concentrations.

**Fig 4:**
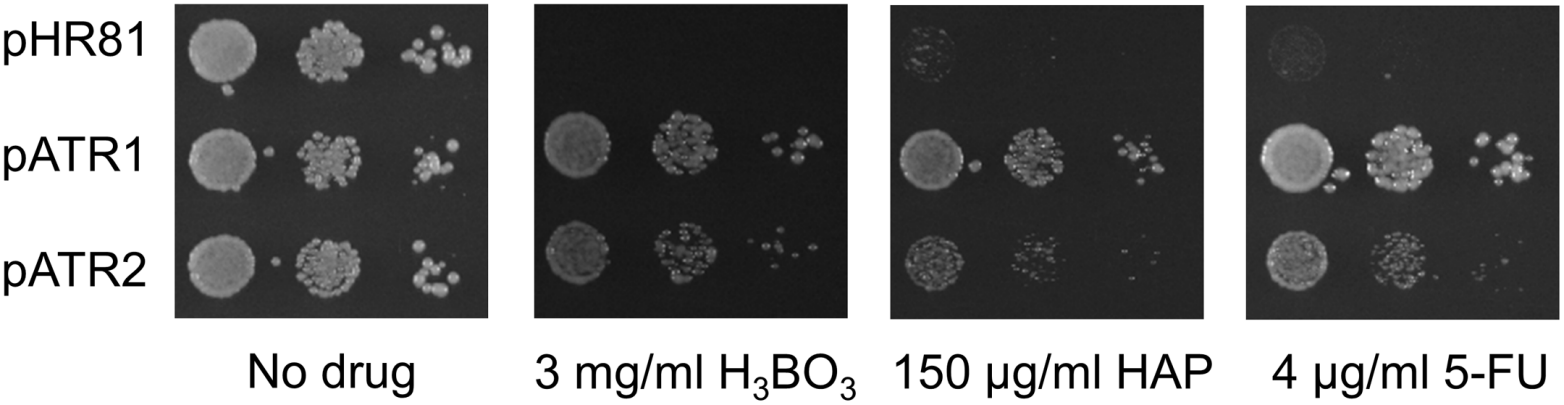
Resistance to HAP, 5-FU and boric acid conferred by overexpression of *ATR1* and *ATR2*. The cloned *ATR1* plasmid and a PCR-amplified *ATR2 (YMR279C)* gene cloned into the vector pHR81 were tested for resistance to HAP, 5-FU and boric acid. Cells transformed with pHR81 [30] were included as a negative control. Transformants were grown in liquid medium to late exponential phase, serially diluted, and then spotted onto uracil- and adenine-less SC galactose (Gal-UA) plates with or without HAP, 5-FU or boric acid at the indicated concentrations.

*ATR1*, finally, encodes a multidrug efflux pump that belongs to the major facilitator superfamily (MFS). Atr1p was originally named from the fact that it is required for resistance to the heterocyclic amine aminotriazole, and *ATR1* expression has been found to increase during DNA-replication stress [38–39]. We found that overexpression of *ATR1* confers strong resistance to both HAP and 5-FU (Fig 2A and 2B). *ATR1* has an unnamed paralogue, *YMR279C*, which similarly to *ATR1* was shown to confer resistance to boric acid [40]. We therefore also tested overexpression of a PCR-cloned copy of that gene (Fig 4). We found that *YMR279C* confers resistance to boric acid, HAP and 5-FU when overexpressed, though not as strongly as *ATR1*. We conclude from this that *YMR279C* has a similar multidrug efflux pump activity as *ATR1*. We therefore propose the name *ATR2* for the open reading frame *YMR279C*.

### Some 5-FU resistance genes also mediate resistance to HAP when overexpressed

Our finding that one of the HAP resistance genes, *ATR1*, also confers resistance to 5-FU prompted us to test if any of our previously identified 5-FU resistance genes [16] would confer resistance to HAP. As shown in Fig 2, we found that overexpression of *HAM1* and *LOG1 (YJL055W)* confers a comparatively strong resistance to HAP and that overexpression of *HMS1* confers a very weak but still detectable HAP resistance (Fig 2A). In contrast, overexpression of the carbamoyl phosphate synthetase subunits *CPA1* or *CPA2* had no apparent effect on the resistance to HAP. Two of the genes that we previously found to confer 5-FU resistance, *HMS1* and *LOG1* [16], did not appear in our screen even though they do confer HAP resistance when overexpressed (Fig 2A). This might suggest that the screen was not exhaustive, though we did recover *HAM1* five times. However, *HMS1* has a very weak effect, and may therefore have escaped detection in our screen.

To assess the strength of the different resistance genes, we examined the size of single cell clones at the appropriate dilution in the presence of HAP or 5-FU, since colony size is a sensitive measure of growth rate in yeast. We found that *HAM1* and *ATR1* have the strongest effect on HAP resistance, followed by *LOG1* and *DUT1*, and then by *ADE4*, *APT2* and *HMS1* in decreasing order of resistance (Fig 2A). This was determined on galactose media since HAP resistance is much easier to score on galactose than on glucose. As for 5-FU resistance, we found that *CPA1* and *ATR1* have the strongest effect, followed by *HAM1*, and then by *HMS1*, *CPA2* and *LOG1* in decreasing order of resistance. This was determined on glucose (Fig 2B). However, on galactose we found that *LOG1* has a much stronger effect, comparable to that of *HAM1*, and that *DUT1* also had a barely but still detectable effect (Fig 2B). The reason why *LOG1* is much more efficient in conferring resistance against 5-FU on galactose remains to be determined. None of the other 5-FU resistance genes showed a similar effect, so it is likely not due to differences in the uptake or metabolism of 5-FU on galactose compared to glucose. A likely explanation for this effect is that *LOG1* is moderately (2–3 fold) repressed by glucose [41].

In the case of *HAM1*, a likely explanation for the resistance to HAP is suggested by its known enzymatic activity. Ham1p has been found to be a nucleoside triphosphate pyrophosphatase, which does not seem to discriminate between deoxyribonucleotides and ribonucleotides. It has the highest specificity against deaminated purines, *e*.*g*. (d)XTP and (d)ITP, amongst the nucleotides assayed, but also showed some residual activity against dATP and dCTP [18]. This suggests that the molecular mechanism by which overexpression of *HAM1* confers resistance to HAP, and likely also to 5-FU, is its pyrophosphatase activity.

The function of the *YJL055W* gene is not known. However, a BLAST search [42] with the Yjl055wp amino acid sequence as probe identified the plant LOG (LONELY GUY) family of proteins as its closest homologues, with E-values of 4e-56 for the rice LOG protein and 2e-52 for the *Arabidopsis* LOG1 protein. *YJL055W* is the only yeast gene encoding a protein with strong similarity to the plant LOG proteins. This suggests that *YJL055W* is an ortholog of the plant *LOG* genes, and we will therefore refer to it as the yeast *LOG1* gene. The plant LOG proteins were originally identified as enzymes that activate adenylate-type pre-cytokinins, a group of plant hormones, by cleaving off the N6-modified adenine nucleobase from the precursor cytokinin nucleoside monophosphate [26,43]. It is therefore conceivable that yeast Log1p may catalyze a similar reaction in the degradation of non-canonical nucleotides (see Discussion).

### Sensitivity of knockout mutants lacking resistance genes to HAP and 5-FU

Overexpression and loss of a gene frequently has opposite effects on the affected phenotypes. In order to determine if this is true in our case, we proceeded to test knockout mutants lacking each of the resistance genes for sensitivity to both HAP and 5-FU. These experiments were carried out using strains transformed with the empty vector pHR81 to facilitate comparisons with the overexpression experiments and avoid complications due to the fact that the genetic background used, BY4742, is *ura3* and thus deficient for pyrimidine biosynthesis, which in turn affects 5-FU sensitivity [16]. The pHR81 plasmid carries the *URA3* marker which restores a functional pyrimidine biosynthesis in these strains. Since the *DUT1* gene is essential, the effect of a haploid knockout mutant could not be tested, but we instead included a heterozygous *dut1/DUT1* strain and a wild type diploid control in order to examine the effect of haploinsufficiency.

**Fig 5:**
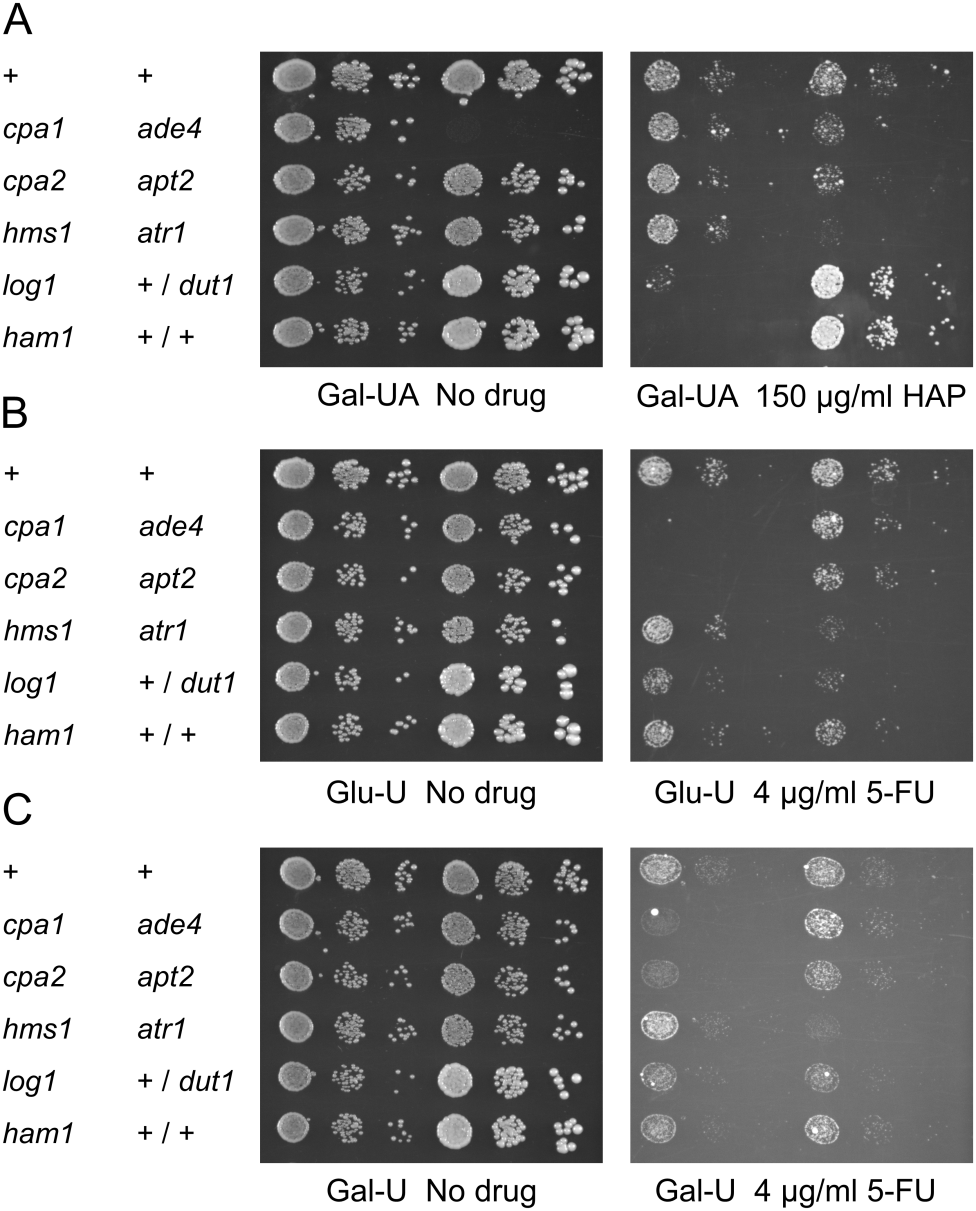
Sensitivity to HAP and 5-FU due to disruption of different resistance genes. Yeast strains disrupted for the genes isolated in our HAP resistance screen and in our previous screen for 5-FU resistance [16] were tested for increased sensitivity to HAP (A) and 5-FU (B and C). All strains were transformed with the empty vector pHR81 in order to complement the *ura3* mutation in the genetic background and thus restore a functional pyrimidine biosynthesis. The + sign stands for the haploid wild type control strain BY4742, +/+ stands for the diploid wild type control strain BY4743, and +/*dut1* for the diploid *DUT1/dut1* heterozygote. Transformants were grown in liquid medium to late exponential phase, serially diluted, and then spotted onto uracil- and adenine-less SC galactose plates (Gal-UA) plates, uracil-less glucose (Glu-U) or uracil-less galactose (Gal-U) plates with or without HAP or 5-FU at the indicated concentrations. The no drug control plates were photographed after 3 days and the drug plates after 5 days.

The sensitivity of different strains to 150 μg/ml HAP is shown in Fig 5A. We found that the *ham1* and *atr1* knockouts are highly sensitive to HAP at this concentration. The sensitivity of the *ham1* knockout is consistent with previous findings that *ham1* strains are sensitive to purine analogues [17,23] and with the known role of Ham1p in dephosphorylation of HAPTP. The sensitivity of the *atr1* knockout to HAP suggests that the Atr1p multidrug efflux pump contributes to HAP detoxification under normal conditions, and not only when overexpressed. We further found that the *log1* and *apt2* knockouts are sensitive to 150 μg/ml HAP, though not as strongly as the *ham1* and *atr1* knockouts (Fig 5A). A sensitivity of the *log1* knockout to purine analogues has been noted previously [23], and is consistent with a proposed role of Log1p in dephosphorylation of nucleoside monophosphates (see Discussion). The sensitivity of the *apt2* knockout to HAP has not been described previously. It suggests that Apt2p contributes to HAP detoxification under normal conditions, and not only when overexpressed. In contrast to these observations, the *cpa1*, *cpa2* and *hms1* strains were not sensitive to HAP (Fig 5A). Nor was the *dut1/DUT1* heterozygote more sensitive to HAP than the wild type diploid. Finally, we note that the sensitivity of the *ade4* strain to HAP could not be assessed since it does not grow in the absence of adenine, a competitive inhibitor of HAP toxicity which must be omitted in order to score sensitivity to HAP. In fact, the *ade4* knockout grows weakly in the presence but not the absence of HAP (Fig 5A). A likely explanation is that deamination of HAP to hypoxanthine permits the *ade4* strain to grow in the absence of adenine.

The sensitivity of different strains to 4 μg/ml 5-FU is shown in Fig 5B (on glucose) and 3C (on galactose). As we previously noted [16], the *cpa1* and *cpa2* knockouts are highly sensitive to 5-FU, but also the *atr1* knockout, which is consistent with a role for the Atr1p multidrug efflux pump in detoxification of 5-FU under normal conditions and not only when overexpressed. Consistent with our previous observations [16], the *log1* knockout was weakly sensitive to 5-FU. Finally, we note that the wild type diploid is more sensitive to 5-FU than the haploid strains, and that this sensitivity is further increased in the *dut1/DUT1* heterozygote. This is a striking effect since diploid strains normally grow much better than the haploids, as evident from the no drug control (Fig 5B). It suggests that diploids are more sensitive to 5-FU than haploids, and also that Dut1p is important for resistance to 5-FU under normal conditions.

### Cross-dependencies between genes that mediate resistance to HAP or 5-FU

**Fig 6:**
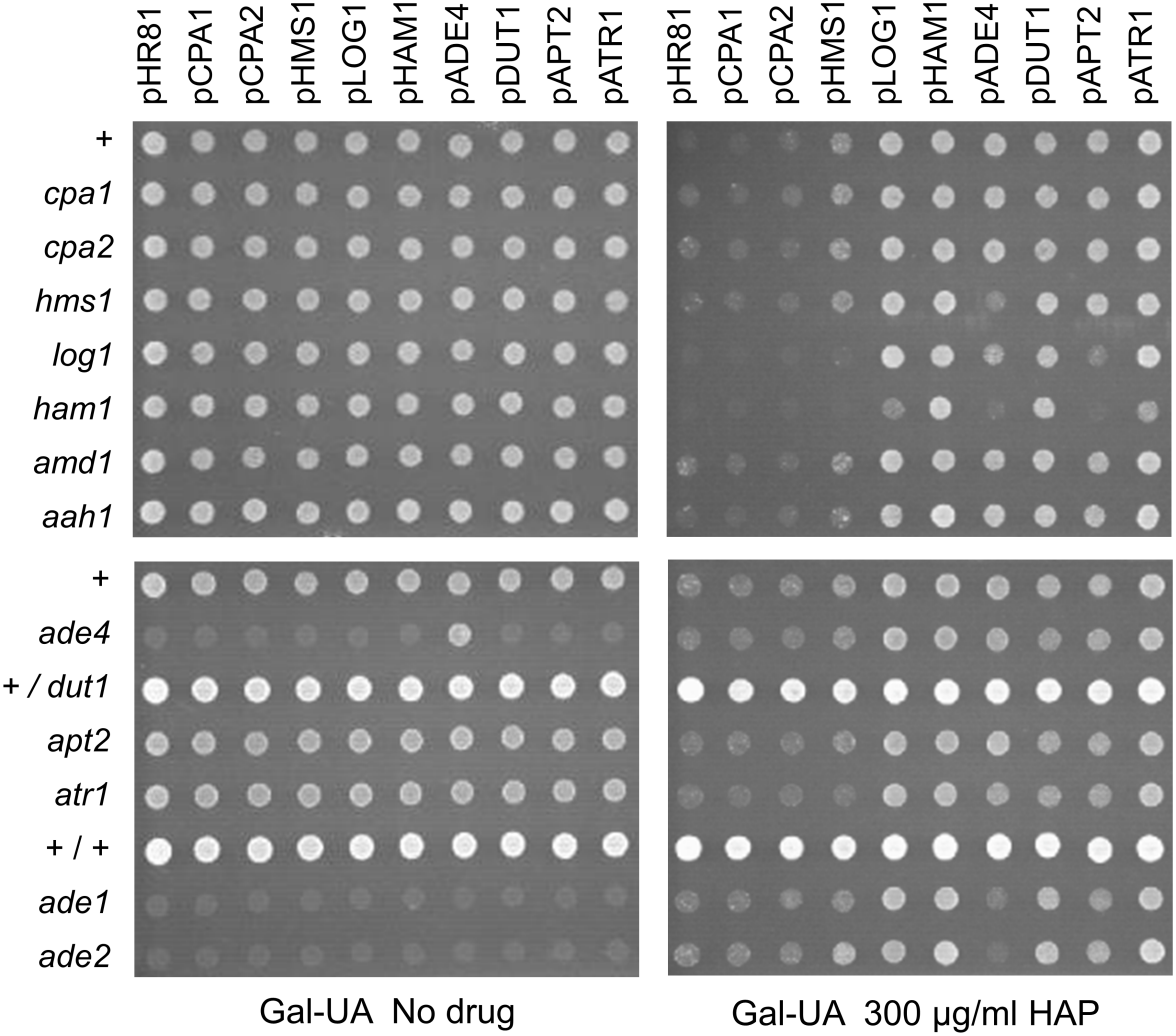
Cross-dependencies between different genes for the ability to confer resistance to HAP when overexpressed. Each plasmid was transformed into yeast knockout strains where one of the other resistance genes had been deleted. Overexpression plasmids (see Fig 1) are shown at the top, yeast strains at the left, and drug concentrations at the bottom in each subfigure. Transformants were grown in liquid medium to late exponential phase, diluted, and then spotted onto uracil- and adenine-less SC galactose (Gal-UA) plates with or without 300 μg/ml HAP. Note that the *ade4*, *ade1*, and *ade2* strains do not grow on adenine-less media in the absence of the drug, but are able to grow weakly in the presence of HAP which is partially deaminated to adenine. The + sign stands for the haploid wild type control strain BY4742, +/+ stands for the diploid wild type control strain BY4743, and +/*dut1* for the diploid *DUT1/dut1* heterozygote. The control vector is pHR81 [30]. The no drug control plate was photographed after 3 days and the HAP plate after 5 days.

**Fig 7:**
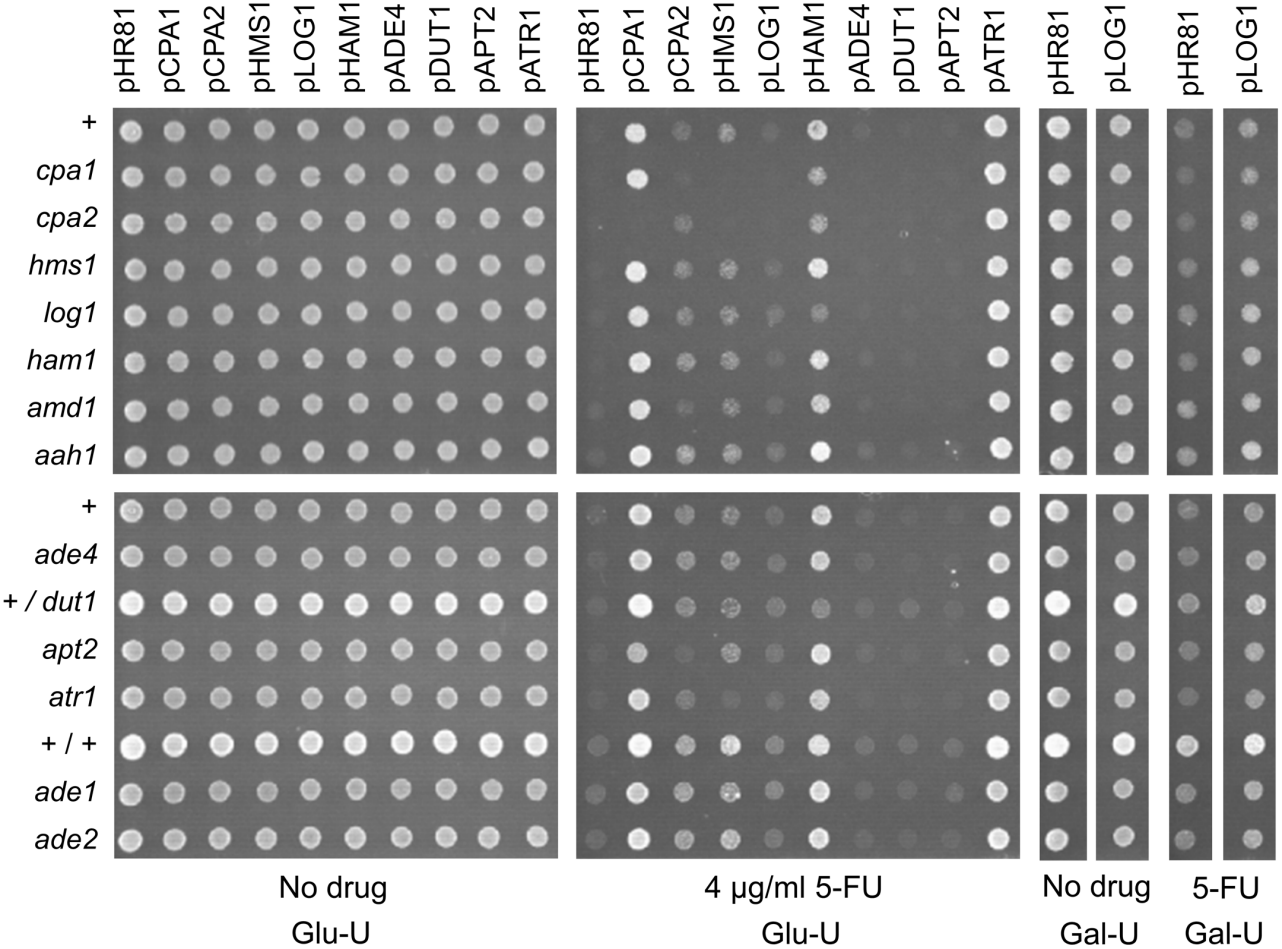
Cross-dependencies between different genes for the ability to confer resistance to 5-FU when overexpressed. Each plasmid was transformed into yeast knockout strains where one of the other resistance genes had been deleted. Overexpression plasmids (see Fig 1) are shown at the top, yeast strains at the left, and drug concentrations at the bottom in the figure. Transformants were grown in liquid medium to late exponential phase, diluted, and then spotted onto uracil-less SC glucose (Glu-U) plates with or without 4 μg/ml 5-FU. Also shown to the right are results obtained on uracil-less SC galactose (Gal-U) plates for the *LOG1* gene and the pHR81 vector control. The + sign stands for the haploid wild type control strain BY4742, +/+ stands for the diploid wild type control strain BY4743, and +/*dut1* for the diploid *DUT1/dut1* heterozygote. The control vector is pHR81 [30]. The no drug control plate was photographed after 3 days and the 5-FU plate after 5 days.

We next tested for genetic interactions and cross-dependencies between the different drug resistance genes by transforming each resistance plasmid into yeast strains where one of the other genes had been knocked out (Figs 6 and 7). The most pronounced genetic interactions affecting HAP sensitivity were seen in the *ham1* knockout. Thus, overexpression of *ADE4*, *APT2* and *HMS1* failed to cause HAP resistance in the *ham1* strain, and the effects of *ATR1* and *LOG1* overexpression were significantly reduced (Fig 6). This suggests that these genes to some extent are dependent on *HAM1* for their resistance phenotype, though it could be argued that the increased HAP sensitivity of the *ham1* knockout would make it difficult to detect the effect of other genes, particularly in the case of *HMS1* which has a rather weak effect. Perhaps more surprisingly, the ability of *DUT1* to confer HAP resistance when overexpressed does not seem to be affected by the *ham1* knockout (Fig 6). We conclude that the HAP resistance conferred by *DUT1* overexpression is strong enough to fully compensate for the increased sensitivity of the *ham1* strain. This somewhat surprising since Ham1p targets both HAPTP and dHAPTP whereas Dut1p is thought to be specific for deoxyribonucleotides. It suggests that the main cytotoxic effect of HAP is mediated by dHAPTP (see Discussion). We further note that *ADE4* overexpression was less efficient in conferring HAP resistance also in the *hms1* and *log1* knockouts. *APT2* overexpression, finally, was also less efficient in conferring HAP resistance in the *log1* knockout (Fig 6).

**Fig 8:**
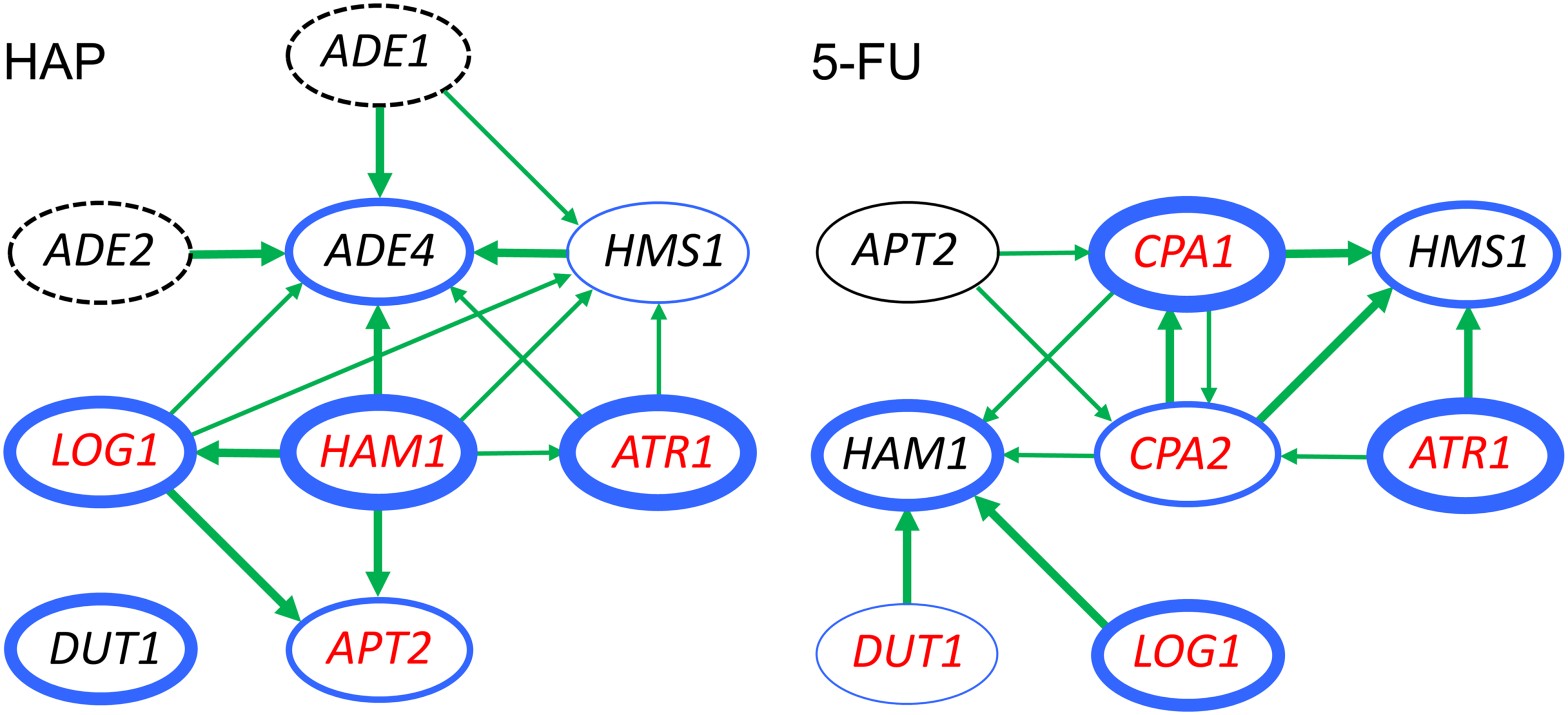
Overview of the genetic interactions observed between different drug resistance genes. Interactions affecting the sensitivity to HAP are shown at the top and those affecting the sensitivity to 5-FU at the bottom. Genes that cause resistance to the drug when overexpressed are enclosed in *blue* ovals, the thickness of which indicates the strength of the effect. The dashed ovals around *ADE1* and *ADE2* means that these genes were not tested for overexpression effects. A *green* arrow between two genes means that the target needs the source in order to confer full resistance when overexpressed, with thick arrows indicating a strong or complete dependence and thin arrows a weaker effect. Genes for which the knockout mutant shows increased sensitivity to the drug are shown in *red*.

Resistance to 5-FU was tested on glucose media and also on galactose media for *LOG1*, since we found that the effect of *LOG1* on 5-FU resistance is much stronger on galactose whereas other effects are more easily seen on glucose. We saw that *CPA1* depends on *CPA2* and *CPA2* depends on *CPA1* (Fig 7), consistent with our previous findings [16]. Interestingly, *HMS1* overexpression depends on *ATR1* for its ability to confer 5-FU resistance, and *HAM1* depends partially on *LOG1* and *DUT1*, and also more weakly on *CPA1* and *CPA2* (Fig 7). Furthermore, *HMS1* also seems to be dependent on *CPA1* and *CPA2* (Fig 7). However, it should be noted that the weak 5-FU resistance conferred by *HMS1* overexpression could be hard to detect in the *cpa1* and *cpa2* strains, which are quite sensitive to 5-FU. Surprisingly, *CPA1* and *CPA2* were also partially dependent on *APT2* (Fig 7). This finding was unexpected since *APT2* was cloned due to its effect on HAP resistance, and since its sequence similarity to *APT1* suggests a role purine metabolism. It is conceivable that this effect may reflect some kind of cross-talk between purine and pyrimidine metabolism, but other explanations are also possible. The observed cross-dependencies for resistance to HAP and 5-FU are summarized in Fig 8.

### Dependencies of resistance genes on other genes involved in purine metabolism

We also tested if knockouts of other genes involved in the purine metabolism would affect the ability of the cloned genes to confer resistance to either HAP (Fig 6) or 5-FU (Fig 7). To this end, we transformed all resistance plasmids into the purine deaminase strains *amd1* (AMP deaminase), and *aah1* (adenine deaminase) to test if the HAP resistance conferred by overexpression of different genes was dependent on the ability to remove, through deamination, the hydroxylamine group of HAP, a reaction that might be performed by adenine deaminases [44]. Finally, we transformed all resistance plasmids into the *ade1* and *ade2* strains to test if resistance was dependent on a functional purine biosynthesis pathway. This experiment was prompted by our finding that the 5-FU resistance conferred by overexpression of *CPA1* and *CPA2* is dependent on the pyrimidine biosynthesis pathway [16].

As shown in Fig 6, we found that the sensitivity to HAP was not significantly affected in the *amd1* and *aah1* knockout strains. Nor were any obvious interactions between *amd1* or *aah1* and any of the other genes conferring HAP resistance seen. However, the HAP resistance conferred by *ADE4* overexpression was abolished in the *ade1* and *ade2* strains (Fig 6). This is consistent with the known function of *ADE4* which encodes phosphoribosylpyrophosphate amidotransferase, the first enzyme in the purine biosynthetic pathway, and supports the notion that *ADE4* overexpression reduces the toxicity of HAP by diluting it with freshly synthesized purines. There was also a small effect of the *ade1* knockout on the HAP resistance conferred by *HMS1* overexpression (Fig 6). The HAP resistance conferred by the *LOG1*, *HAM1*, *DUT1*, *APT2* and *ATR1* genes were all unaffected by the *ade1* and *ade2* knockouts, and thus apparently independent of *de novo* purine biosynthesis. As expected, the *amd1*, *aah1*, *ade1* and *ade2* knockouts had no effects on the resistance to 5-FU in any of the strains (Fig 7).

### Effect of resistance genes on the sensitivity to other purine and pyrimidine analogues

**Fig 9:**
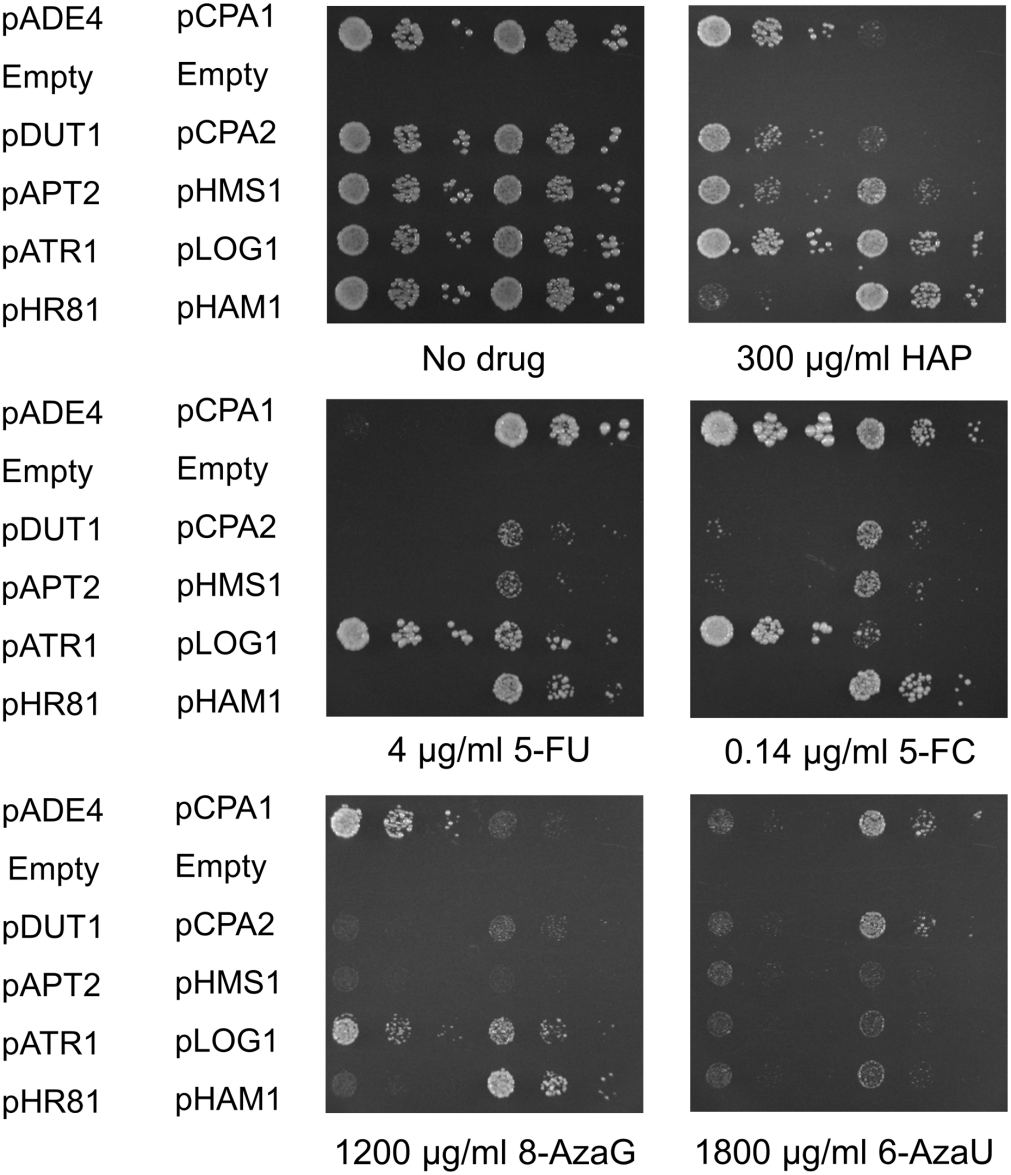
Resistance to 5-FC, 6-azauracil and 8-azaguanine conferred by overexpression of different genes. The genes isolated in our HAP resistance screen were tested for resistance to 5-FC, 6-AzaU and 8-AzaG. No drug controls are also shown. Also included were the genes isolated in our previous screen for 5-FU resistance [16]. Cells transformed with the empty vector pHR81 [30] were included as a negative control. Transformants were grown in liquid medium to late exponential phase, serially diluted, and then spotted onto uracil- and adenine-less SC galactose (Gal-UA) plates with or without HAP, 5-FU, 5-FC, 6-AzaU or 8-AzaG at the indicated concentrations. An empty row (Empty) was left below the pADE4 and pCPA1 transformants in order to prevent effects on adjacent strains due to the release of adenine and uracil into the media.

In order to test the generality of our findings with HAP and 5-FU, we also tested our cloned resistance genes with several other purine and pyrimidine analogues. A problem with such experiments is that not all drugs are toxic in yeast, due to poor uptake or failure of conversion to the active metabolite. In total, we tested six purine analogues (2-chloroadenine, 6-thioguanine, 6-mercaptopurine, 6-N-hydroxyaminopurine, 2-amino-6-hydroxylaminopurine, and 8-azaguanine) and three pyrimidine analogues (6-azauracil, 5-azacytosine and 5-fluorocytosine). Six of the drugs (2-chloroadenine, 6-thioguanine, 6-mercaptopurine, 6-N-hydroxyaminopurine, 2-amino-6-hydroxylaminopurine, and 5-azacytosine) were not toxic at the highest concentrations that could be tested without precipitation of the drug. The purine analogue 8-azaguanine (8-AzaG) and the pyrimidine analogues 6-azauracil (6-AzaU) and 5-fluorocytosine (5-FC) did show toxicity, and we proceeded to test the effects of the resistance genes on sensitivity to these three drugs. The results are shown in Fig 9, with HAP and 5-FU included as controls.

For the purine analogue 8-AzaG, the pattern was similar to that seen with HAP in that overexpression of *ADE4*, *ATR1*, *LOG1* and *HAM1* all caused resistance to the drug. We presume that the mechanisms involved are similar to those for HAP resistance. However, overexpression of *DUT1*, *APT2* or *HMS1* did not cause any significant resistance to 8-AzaG (Fig 9). For the pyrimidine analogue 6-AzaU, we saw that overexpression of *CPA1* and *CPA2* caused resistance, similar to the case with 5-FU. However, the other plasmids did not have much of an effect. The other pyrimidine analogue, 5-FC, was more similar to 5-FU in its pattern of sensitivity. However, a striking difference is that overexpression of *ADE4* confers resistance to 5-FC but not to 5-FU (Fig 9). This was an unexpected finding since *ADE4* confers resistance to HAP and other purine analogues by boosting the *de novo* synthesis of purines. (Fig 9).

## Discussion

We have performed a screen for genes whose overexpression confer resistance to HAP. To facilitate detection of weak effects, the screen was carried out both in a wild type yeast strain and in a *ham1* knockout strain that has increased sensitivity to HAP [17]. In addition to *HAM1*, we found four genes conferring HAP resistance: *ADE4*, *ATR1*, *DUT1* and *APT2* (Figs 1 and 2). To find out more about the mechanisms by which the genes cause resistance to HAP, we also tested knockout mutants for sensitivity to HAP. Furthermore, we carried out a cross-dependency test in which all plasmids were transformed into strains with knockouts of each one of the other genes, and tested for the abilities to confer HAP resistance (Fig 6). Also included in this experiment were the five genes that we previously found to confer resistance to 5-FU when overexpressed: *CPA1*, *CPA2*, *HMS1*, *LOG1* and *HAM1* [16], and four genes involved in purine *de novo* synthesis and salvage: *ADE1*, *ADE2*, *AMD1*, *AAH1*. All strains and plasmids were also tested for resistance to 5-FU in order to identify common and unique mechanisms of drug resistance. The observed effects and genetic interactions are summarized in Fig 8. We found that four of the genes confer resistance to both HAP and 5-FU: *HMS1*, *LOG1*, *HAM1*, and *ATR1*. *APT2* and *ADE4* only confer HAP resistance, whereas *CPA1* and *CPA2* only confer resistance to 5-FU. *DUT1* mainly confers HAP resistance, though a barely detectable effect was also seen on resistance to 5-FU.

**Fig 10:**
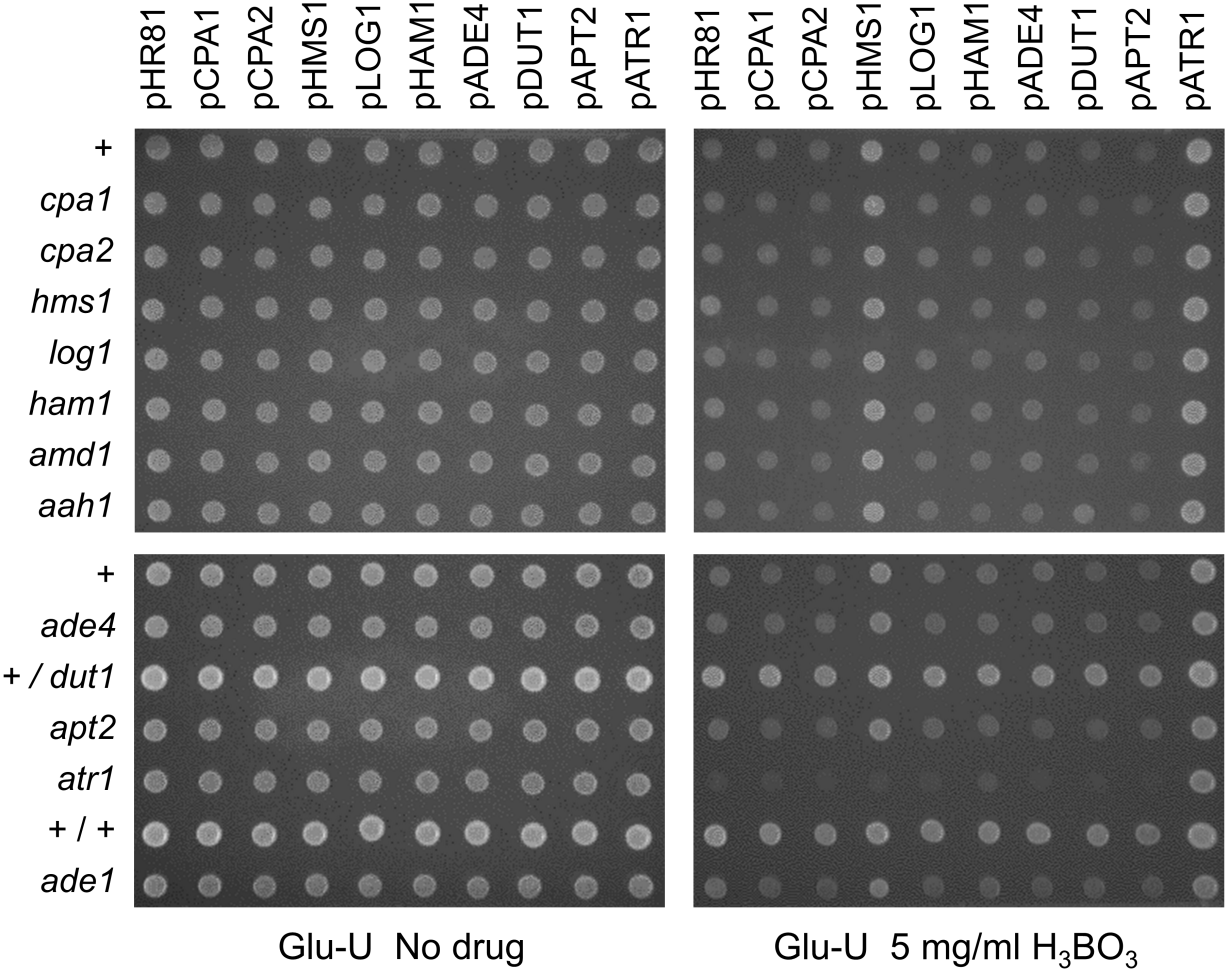
Boric acid sensitivity of the *atr1* knockout strain and boric acid resistance conferred by *HMS1* overexpression. Each plasmid was transformed into yeast knockout strains where one of the resistance genes had been deleted. Overexpression plasmids (see Fig 1) are shown at the top, yeast strains at the left, and drug concentrations at the bottom. Transformants were grown in liquid medium to late exponential phase, diluted, and then spotted onto uracil-less SC glucose (Glu-U) plates with or without 80 mM boric acid (H_3_BO_3_). The + sign stands for the haploid wild type control strain BY4742, +/+ stands for the diploid wild type control strain BY4743, and +/*dut1* for the diploid *DUT1/dut1* heterozygote. The control vector is pHR81 [30]. Note that *HMS1* overexpression has no effect in the *atr1* knockout strain, indicating that Atr1p functions downstream of Hms1p in conferring resistance to boric acid.

The cloned genes represent four different mechanisms by which a cell may acquire resistance to nucleotide analogues. The first resistance mechanism is to promote efflux of the drug, and is exemplified by *ATR1*, *ATR2* and *HMS1*. The *ATR1* gene encodes a multidrug efflux pump, and a likely reason for the strong resistance to both HAP and 5-FU conferred by overexpression of *ATR1* is that Atr1p pumps out the non-canonical nucleotides, (d)HAPMP and 5-F(d)UMP, or the corresponding free bases. *HMS1* encodes a *myc*-related transcription factor that was found to activate *ATR1* expression in a microarray experiment [45]. This provides a likely explanation for our findings that overexpression of *HMS1* confers resistance to both 5-FU and HAP. Consistent with this, we found that *HMS1* has no effect on either 5-FU or HAP resistance in the *atr1* knockout strain (Figs 6 and 7). In further support of this, we found that *HMS1* overexpression confers resistance to boric acid, for which *ATR1* has been shown to be important [46], and that this effect also disappears in the *atr1* knockout strain (Fig 10). The genetic interactions between *ATR1*, *HMS1*, *LOG1* and *HAM1* in their effects on HAP resistance (Fig 6) supports the notion that Atr1p acts as an efflux sink for the products of Ham1p and Log1p. We interpret these interactions as synergisms between drug efflux (Atr1p and Hms1p) and other detoxification mechanisms (Log1p and Ham1p). Finally, our finding that the *ATR1*-related gene *ATR2* (*YMR279C)* also confers resistance to HAP and 5-FU (Fig 4) suggests that its gene product acts as a multidrug efflux pump similar to Atr1p.

The second resistance mechanism is to dilute the drug or its activated metabolites by boosting the *de novo* synthesis of nucleotides. This mechanism is exemplified by *CPA1*, *CPA2* and *ADE4*. *CPA1* and *CPA2* overexpression boosts *de novo* pyrimidine synthesis, thus preventing 5-FU from exerting its toxic action [16]. Overexpression of *ADE4*, which encodes the first enzyme in purine biosynthesis, similarly boosts *de novo* purine synthesis, which dilutes HAP and prevents it from exerting its toxic action. As expected, we found that these resistance genes are highly specific for the type of nucleotide: *CPA1* and *CPA2* do not confer resistance to HAP (Fig 6), and *ADE4* does not confer resistance to 5-FU (Fig 7). However, *CPA1* and *CPA2* do confer resistance to other pyrimidine analogues such as 5-fluorocytosine and 6-azauracil (Fig 9).

**Fig 11:**
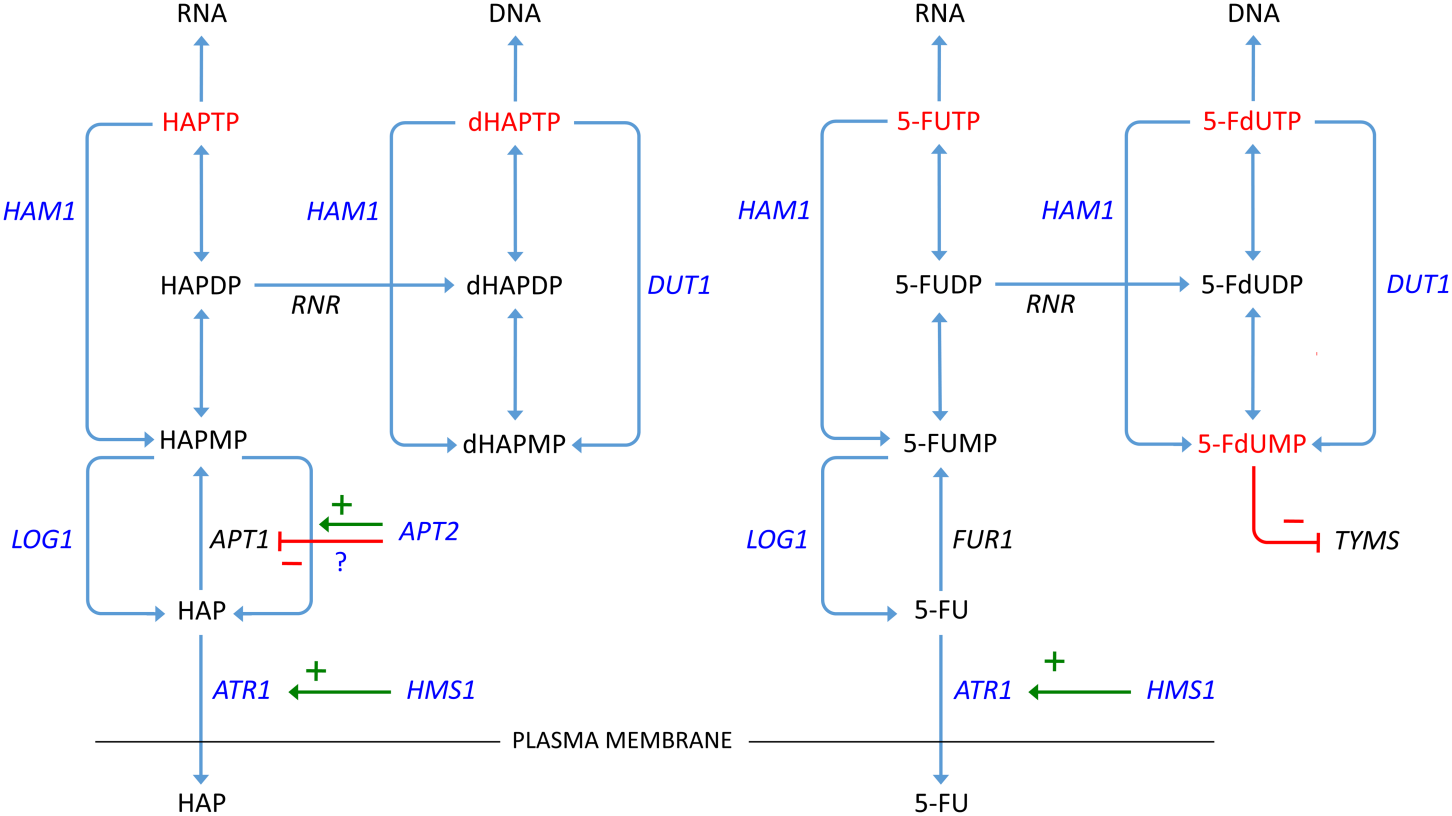
Proposed models for mechanisms of resistance to HAP (left) and 5-FU (right). Gene symbols are shown adjacent to the metabolic reactions proposed to be catalyzed by the encoded proteins. Genes that cause drug resistance when overexpressed are shown in *blue* and activated toxic metabolites of HAP and 5-FU in *red*. Metabolic reactions are shown as *blue* arrows, positive regulatory effects in *green*, and negative regulatory effects in *red*. For *APT2*, two possible effects on HAP metabolism are indicated: either inhibition of *APT1* or catalysis of the opposite reaction, *i*.*e*. conversion of HAPMP to HAP. *RNR* stands for ribonucleotide reductase and *TYMS* for thymidylate synthase. Not shown in the figure are the effects of overexpressing *ADE4* and *CPA1* or *CPA2*, which boost purine and pyrimidine synthesis, and thus dilute HAP and 5-FU, respectively.

A third resistance mechanism is to interfere with activation of the drug, and it is possible that *APT2* could act in this way. Our finding that *APT2* overexpression confers resistance to HAP is surprising since previous studies did not detect a knockout phenotype or enzymatic activity associated with *APT2*, which was therefore proposed to be a pseudogene [37]. The homology to *APT1* which encodes APRT suggests that *APT2*, if active, should encode an enzyme with similar activity. APRT is needed for activation of HAP into its toxic metabolite HAPMP [24], so overexpression of a protein with APRT activity would be expected to make cells more rather than less sensitive to HAP. Consistent with this, we found that overexpression of *APT1* did make the cells slightly more sensitive to HAP (Fig 3). One possible explanation for our finding that overexpression of *APT2* confers resistance to HAP could be that it interferes with the expression of Apt1p and thus with its ability to activate HAP. Such interference could occur by promoter competition for an activator of both genes, since *APT2* is known to be transcribed [37]. Alternatively, since APRT is a dimeric enzyme [37], overexpression of *APT2* might lead to the formation of inactive Apt1p-Apt2p heterodimers. However, a second possible explanation for our finding could be that Apt2p has a previously undetected activity that helps to detoxify HAP. APRT catalyzes a reversible reaction [47], so one possibility is that Apt2p is an APRT that favours the reverse reaction, converting HAPMP to HAP. Yeast isozymes can have opposite favoured directions; one example of this is the alcohol dehydrogenases Adh1p and Adh2p [48]. Our finding that the *apt2* knockout is moderately sensitive to HAP (Fig 5A) is consistent with the second explanation, since it suggests that *APT2* is not just a pseudogene. Our finding that *APT1* overexpression confers resistance to 5-FU (Fig 3) could be due to interference with activation of 5-FU to 5-FUMP (Fig 11), since the 5-FU activating enzyme Fur1p and the adenine activating enzyme Apt1p both use the same substrate, PRPP. Purines have been shown to relieve 5-FU toxicity in a cell line, and it was suggested that this is due to PRPP being depleted during activation by nucleobase phosphoribosyltransferases [49].

The fourth resistance mechanism is to detoxify the drug by degrading it or its activated metabolites. All organisms need to keep their nucleotide pools free from non-canonical nucleotides that might cause damage if incorporated into DNA or RNA. Some non-canonical nucleotides are generated continuously from the metabolism, such as dUTDP produced as an intermediate in TMP synthesis, or IMP and XMP which are intermediates in AMP and GMP synthesis. Other non-canonical nucleotides are generated by oxidation or deamination of canonical nucleotides. Several enzymes have evolved to deal with the threat posed by non-canonical nucleotides [50], and overexpression of such enzymes is expected to confer resistance to nucleotide analogues. This resistance mechanism is exemplified by the *DUT1* and *HAM1* genes and, as argued below, we also think that *LOG1* belongs to this class of genes.

*DUT1* encodes dUTP pyrophosphatase, which degrades the genotoxic dUTP generated from the dUDP produced during TMP synthesis [51]. It is an essential enzyme in many organisms including yeast [52]. Dut1p was long thought to be specific for dUTP, but it was more recently found to have significant activity also against the non-canonical purine nucleotide dITP [52]. Our finding that overexpression of *DUT1* confers resistance to HAP suggest that Dut1p may have an even broader specificity, including purine analogue triphosphates such as dHAPTP. In this context, our finding that overexpression of *DUT1* has little or no effect on the resistance to 5-FU is surprising since 5-FdUTP is more similar to dUTP, and since the *dut1/DUT1* heterozygote, as expected, was sensitive to 5-FU but not to HAP (Fig 5). However, dephosphorylation of 5-FdUTP generates 5-FdUMP which is also highly toxic due to its inhibition of thymidylate synthase [4]. It is conceivable that increased production of 5-FdUMP could explain why overexpression of *DUT1* has little or no beneficial effect on 5-FU toxicity. Since dHAPMP is not toxic, a similar situation would not exist for HAP toxicity, which could explain why *DUT1* overexpression has an effect in that case.

Interestingly, we observed a significantly higher sensitivity to 5-FU in the diploids, both in the wild type and in the *dut1/DUT1* heterozygote (Fig 5B). A likely reason for this is that the response to genotoxic stress differs between haploids and diploids. Thus, Li and Tye [53] found that the replication stress induced by a defective *mcm4* allele caused a diploid-specific severe genetic instability and reduced viability. It was suggested that this is due to different repair pathways being favoured in haploid and diploid cells. In haploids, replication stress mainly induces Rad6-dependent pathways that resume stalled forks, whereas diploids use the Rad52- and MRX-dependent pathways that repair double strand breaks. Presumably, the latter type of repair events are lethal when massively induced, which may explain both the reduced viability of *mcm4* diploids [53] and our finding that diploids are more sensitive to 5-FU than haploids (Fig 5B).

The *HAM1* gene was discovered in a screen for yeast genes that cause increased sensitivity to HAP when mutated [17]. Ham1p and its orthologues in other species are purine nucleoside triphosphate phosphatases with specificity for (d)ITP and (d)XTP [18,54–55]. This suggested that Ham1p has evolved to deal with the threat posed by these naturally occurring non-canonical purine nucleotides. However, it was subsequently shown that overexpression of *HAM1* also confers resistance to the pyrimidine analogues 5-bromodeoxyuridine [56] and 5-FU [16]. This indicates that the Ham1p enzyme has a broader specificity, being active also against non-canonical pyrimidine nucleotides. Our finding that overexpression of *DUT1* is fully able to compensate for the increased HAP sensitivity of the *ham1* strain (Fig 6) is interesting, as Dut1p is thought to be specific for deoxyribonucleotides, and would thus presumably target only dHAPTP but not HAPTP. It suggests that the genotoxic effects of dHAPTP are more important for HAP toxicity than the effects of HAPTP incorporation into RNA.

Our finding that *LOG1* confers resistance to HAP when overexpressed is consistent with the previous finding that a *log1* knockout is sensitive to HAP [23]. The function of the yeast Log1 protein remains to be determined. However, its homology to the plant LOG proteins, which produce free cytokinins by cleaving off the N6-modified adenine from cytokinin nucleoside monophosphates [26,43], is intriguing since it is the same N6-position of adenine that is modified in HAP. It is therefore likely that Log1p confers resistance to HAP by cleaving off the HAP nucleobase from the activated (deoxy)ribonucleoside monophosphate, (d)HAPMP, thus preventing its conversion into (d)HAPTP that can be incorporated into RNA or DNA. This would be consistent with the partial dependence of *LOG1* on *HAM1* for its ability to confer HAP resistance when overexpressed (Fig 6), since Log1p would then function downstream of Ham1p in the degradation of non-canonical purine nucleotides (Fig 11). The fact that *LOG1* confers resistance to 5-FU when overexpressed further suggests that Log1p is active also against non-canonical pyrimidine nucleoside monophosphates such as 5-FUMP. The partial dependence of *HAM1* on *LOG1* for the ability to confer 5-FU resistance (Fig 7) is consistent with this notion.

In order to assess how general our findings were, we also tested the effects of the cloned resistance genes on the sensitivity to nine additional purine and pyrimidine drugs. Six of the drugs failed to produce any toxicity in yeast, but results were obtained with the pyrimidine analogues 5-FC and 6-AzaU and the purine analogue 8-AzaG, which are shown in Fig 9.

The resistance gene profile of 8-AzaG was similar to that of HAP, except for the fact that overexpression of *DUT1*, *APT2* and *HMS1* did not confer any significant resistance to 8-AzaG. The absence of an effect of *DUT1* is likely due to 8-AzaG toxicity being mainly caused by its incorporation into RNA, which inhibits protein synthesis [57]. Hence, a reduction in any 8-aza-dGTP formed is not expected to relieve toxicity. The absence of an effect of *APT2* is likely due to the fact that activation of guanine and 8-AzaG to ribonucleotides is catalyzed by hypoxanthine-guanine phosphoribosyltransferase, encoded by the yeast *HPT1* gene [58], in contrast to HAP, which is mainly activated by *APT1* [24]. The lack of a detectable effect of *HMS1* on 8-AzaG toxicity could simply be due to the fact that *HMS1* was the weakest resistance gene recovered in our screen (Fig 2A).

The resistance gene profile of 6-AzaU was more narrow than that of 5-FU. Thus, we saw a significant effect only with the *CPA1* and *CPA2* genes (Fig 9). A likely explanation for this is that unlike 5-FU, 6-AzaU does not get incorporated into RNA or DNA, but exerts its toxic effect by inhibiting the pyrimidine biosynthetic enzyme OMP-decarboxylase [57]. It is therefore not surprising that overexpression of *CPA1* and *CPA2* which boosts pyrimidine biosynthesis and thus provides more substrate for OMP-decarboxylase can relieve the 6-AzaU toxicity.

The resistance gene profiles of 5-FC and 5-FU were similar, but interestingly, overexpression of *ADE4*, which boosts *de novo* synthesis of purines, confers resistance to 5-FC but not 5-FU (Fig 9). A likely explanation is that the uptake of 5-FC and 5-FU in yeast is mediated by different transporters. Uracil and 5-FU are taken up by the uracil permease Fur4p, which is feedback-inhibited by intracellular pyrimidines [59–61]. 5-FC is instead taken up by the purine and cytosine permease Fcy2p, which is not inhibited or repressed by cytosine [59], but possibly by an adenine metabolite [62]. Furthermore, *ADE4* overexpression has been shown to cause excretion of inosine and hypoxanthine [63], and hypoxanthine acts as a competitive inhibitor of Fcy2p mediated cytosine uptake that can also relieve 5-FC toxicity [60,62]. We conclude that the resistance to 5-FC conferred by *ADE4* overexpression most likely is due to its effect on Fcy2p-mediated uptake of 5-FC.

In conclusion, it seems that Ham1p, Log1p and Dut1p all have broader specificities than initially thought, affecting both purines and pyrimidines (Fig 11). Together, they serve as gatekeepers that prevent non-canonical bases from being incorporated into nucleic acids, by dephosphorylating nucleoside triphosphates (Ham1p and Dut1p) and by cleaving the resulting nucleoside monophosphates into free bases and ribose-1-phosphate (Log1p). Ham1p targets both ribo- and deoxyribonucleoside triphosphates, thereby preventing the incorporation of non-canonical bases into RNA and DNA. Dut1p only dephosphorylates deoxyribonucleoside triphosphates. It may have evolved to deal with the special threat posed by the genotoxic dUTP generated during biosynthesis of TTP, but also targets non-canonical purine deoxyribonucleotides such as dITP and dHAPTP. Log1p, finally, acts downstream of Ham1p by cleaving its products, which should facilitate the Ham1p reaction by keeping the concentrations of these products low, and prevent reactivation by phosphorylation. Based on the wide phylogenetic distribution of the LOG (LONELY GUY) family of enzymes, it seems likely that keeping the nucleotide pool free from non-canonical nucleotides is their original function, and that their role in cytokinin production in plants and some microorganisms [26–28] is a more recent development in these organisms.

## Abbreviations

5-FC: 5-fluorocytosine
5-FU: 5-fluorouracil
6-AzaU: 6-azauracil
8-AzaG: 8-azaguanine
HAP: 6-N-hydroxylaminopurine
ITP: inosine triphosphate
PRPP: phosphoribosyl pyrophosphate
SC: synthetic complete media
TYMS: thymidylate synthase
XTP: xanthosine 5'-triphosphate
